# Effect of type 2 diabetes mellitus on sperm quality and outcomes of assisted reproductive techniques in infertile male patients

**DOI:** 10.3389/fendo.2025.1692746

**Published:** 2026-01-05

**Authors:** Rui Wang, Yajuan Zhang, Hui Li, Wanpeng Liu, Lin Xu, Meisong Lu

**Affiliations:** Department of Reproductive Medicine, The First Affiliated Hospital of Harbin Medical University, Harbin, China

**Keywords:** male infertility, type 2 diabetes mellitus, sperm, IVF, ICSI

## Abstract

**Background:**

Diabetes mellitus (DM) is one of the most common endocrine disorders affecting various physiological systems and tissues, including the reproductive organs in men. However, the consequences of DM for male individuals undergoing assisted reproductive technology (ART) remain unclear.

**Objective:**

To investigate the sperm quality and the outcomes of *in vitro* fertilization - embryo transfer (IVF-ET) and intracytoplasmic sperm injection - embryo transfer (ICSI-ET) in patients with type 2 diabetes mellitus (T2DM).

**Methods:**

We retrospectively analyzed data from 318 infertile couples for IVF/ICSI cycle outcomes. The experimental group included 106 infertile couples (with male partners all diagnosed with T2DM) who underwent ART treatment at our center. The negative control group consisted of 212 non-diabetic infertile couples, matched by male/female body mass index (BMI) (± 1 kg/m²), male/female age (± 2 years), and serum anti-Müllerian hormone (AMH) levels (± 2 ng/mL). Patients were categorized into two groups based on clinical pregnancy. The χ² test and logistic regression analysis were used to identify and determine the influencing factors of clinical pregnancy.

**Results:**

Infertile men with T2DM had significantly lower sperm volume (2.4 ± 2.2 vs. 2.7 ± 1.2, P < 0.01) and significantly decreased sperm motility compared to the control group (23.0 ± 17.2 vs. 29.7 ± 15.3, P < 0.01). In IVF/ICSI cycles, both groups exhibited similar results in two-pronuclear (2PN) fertilization rates and high-grade embryo rates (*p* > 0.05). The implantation rate, clinical pregnancy rate, and miscarriage rate also showed no significant differences (*p* > 0.05).

**Conclusion:**

T2DM in infertile men exerts adverse effects on sperm quality; however, the decline in sperm quality did not translate into compromised clinical ART outcomes.

## 1 Introduction

Approximately 10% of couples worldwide suffer from infertility, with the male factor accounting for half of the cases (1). Over the past few decades, semen parameters commonly used to assess male fertility have notably declined. This decline can be attributed to various factors, such as unhealthy lifestyle habits, excessive stress, environmental pollutants, and related diseases. Of particular concern is the increasing prevalence of overweight and obesity among individuals of reproductive age. These two weight-related conditions are often accompanied by hyperglycemia, hyperlipidemia, and hypertension, which significantly raise the risk of impaired male fertility (2, 3).

Diabetes mellitus (DM) is a disease that has attracted widespread attention as a public health issue. Among individuals with DM, 90%–95% are diagnosed with type 2 diabetes mellitus (T2DM), which is characterized by insulin resistance, relative insulin underproduction, and elevations in fasting and postprandial glycemia (4, 5). In the long term, diabetes can trigger a series of complications such as cardiovascular disorders, peripheral nerve injury, and reproductive system dysfunction (6, 7).

China holds the largest number of diabetic patients in the world. Since 2000, the growth rate of T2DM cases has significantly accelerated and shown a trend toward younger onset, with the number of male patients being slightly higher than that of female patients (8). Extensive research has investigated the impact of T2DM on female fertility and the reproductive system (9), but its effects on male reproductive function remain unclear. The majority of studies have reported that semen parameters—including volume, concentration, count, motility, and morphology—are generally lower in diabetic patients are generally lower than those in non-diabetic individuals (10). Thus, diabetes is considered a significant risk factor for male infertility; however, the corresponding pregnancy outcomes and underlying mechanisms remain somewhat controversial (11, 12).

Previous reports have proposed several possible pathways. Elevated blood glucose can increase excessive reactive oxygen species (ROS) production in semen via glucose auto-oxidation, polyol pathway activation, and mitochondrial dysfunction, leading to an increased sperm DNA fragmentation index (DFI). In addition, accumulated advanced glycation end-products (AGEs) decrease sperm motility and increase deformity rates. Hyperglycemia may also cause multiple mitochondrial injuries, resulting in insufficient ATP production in sperm. Furthermore, diabetes may impair the function of seminiferous tubules and testicular cells by negatively affecting the hypothalamic–pituitary–gonadal axis and may impact spermatogenesis by disrupting the glucose metabolism process (13–15).

Clinical data indicate that patients with T2DM have a higher risk of infertility (Bener et al. reported an infertility rate of 35.1% among 857 T2DM patients) (16). Many men with T2DM are likely to seek assisted reproductive technology (ART) due to severe declines in semen quality. However, few studies have investigated the correlation between patients with T2DM and ART outcomes. For instance, reduced sperm motility may decrease the ability to penetrate the zona pellucida, and high DFI may lead to fertilization failure, embryonic developmental arrest, and an increased risk of early miscarriage (17, 18).

This study aims to provide potential clinical guidance through an overall assessment of semen parameters in male patients with T2DM, along with pregnancy outcomes during ART treatment.

## 2 Materials and methods

### 2.1 Patients

This retrospective cohort study included 318 infertile couples who underwent their first complete fresh *in vitro* fertilization–embryo transfer (IVF-ET) or intracytoplasmic sperm injection–embryo transfer (ICSI-ET) cycle from January 2018 to September 2024, and it was undertaken in the Department of Reproductive Medicine at the First Affiliated Hospital of Harbin Medical University. This study was reviewed and approved by the Ethics Committee of the First Affiliated Hospital of Harbin Medical University (2025JS40). The experimental group consisted of 106 couples with T2DM-positive husbands (previous diagnosis of T2DM) and T2DM-negative wives. Individuals in the control group were randomly matched by male/female body mass index (BMI) (± 1 kg/m²), male/female age (± 2 years), and serum anti-Müllerian hormone (AMH) levels (± 2 ng/mL) at a ratio of 1:2. Both husbands and wives in the control group were T2DM-negative. The study design was similar to that previously described (19).

Infertility was defined as the inability to achieve a clinically recognized pregnancy after at least 12 months of attempts, and served as the inclusion criterion for this study (20). Patients were excluded if they met any of the following criteria: a diagnosis of type 1 diabetes, other specific types of diabetes mellitus or other forms of glycemic abnormalities, diabetes-related complications, chromosomal abnormalities, varicocele, long-term drug use, HIV seropositivity, a surgical history, or congenital defects (urological or related to reproductive). A total of 106 infertile men with T2DM were included in the experimental group, and a reference population of 212 individuals who had never been diagnosed with any history of glycemic disorders was included in the control group.

### 2.2 Semen analysis

Semen samples obtained from the infertile male patients were collected by masturbation after 2–7 days of sexual abstinence. All samples were analyzed after liquefaction for 30 minutes at 37 °C. Sperm volume, pH, concentration, motility, and morphology were evaluated according to the 1999 WHO (World Health Organization) guidelines. Asthenozoospermia was defined as rapid progressive motility (a) <25% or progressive motility (a + b) <50%. The analysis methods followed the Joint European Society of Human Reproduction and Embryology–Nordic Association for Andrology (ESHRE– NAFA) manual (21).

### 2.3 Ovarian stimulation protocols

Controlled ovarian hyperstimulation (COH) protocols were selected based on the patient’s age, BMI, ovarian reserve, and previous medical history. In the early-follicular GnRH agonist long protocol, a long-acting GnRH agonist (GnRH-a, Leuprorelin acetate, Beiyi; 3.75 mg; Shanghai Livzon Pharmaceutical Co., Ltd., China) was administered on days 2–4 of menstruation. Follicles were monitored via transvaginal ultrasound after 28 days, and serum sex hormone levels were measured simultaneously. When the downregulation criteria were achieved (serum LH < 5 IU/L, E2 < 50 pg/mL), a daily dose of 150–300 IU recombinant human follitropin alfa (rFSH, GONAL-f; 75 IU; Merck Serono S.p.A., Italy) was administered and adjusted based on the ovarian response throughout the stimulation process. Human menopausal gonadotropin (HMG, Lebaode; 75 IU; Livzon Pharmaceutical Factory, Zhuhai, China) and recombinant human lutropin alfa (rFSH, GONAL-f; 75 IU; Merck Serono S.A., Switzerland) were judiciously added as needed, based on follicular development, until follicular maturation.

In the luteal-phase GnRH agonist long protocol, a daily dose of 0.1 mg of the short-acting GnRH agonist (Triptorelin acetate, Decapeptyl; 0.1 mg; Ferring GmbH, Germany) was injected in the mid-luteal phase for 10–14 days until the pituitary function was fully downregulated. The subsequent stimulation process was performed as described above.

The GnRH antagonist protocol began with an initial dose of 150–300 IU/day rFSH on days 2–4 of menstruation. A GnRH antagonist (Cetrorelix acetate, Cetrotide, 0.25 mg; Merck Europe B.V., The Netherlands) was administered once at least two follicles reached 13–14 mm or serum LH exceeded 5 IU/L. HMG and rLH were judiciously added as needed until follicular maturation.

In all protocols, follicular development was monitored by transvaginal ultrasound and serum sex hormone measurements. Once at least 2–3 follicles reached a diameter of >18 mm, 250 μg of recombinant hCG (rhCG, Ovidrel, 250 μg; Merck Serono S.p.A., Italy) was administered as a trigger, and oocyte retrieval was performed 36–38 h after the hCG injection. The choice of insemination method (IVF or ICSI) primarily depended on sperm quality and clinical indications.

One or two embryos were transferred on day 3 (cleavage stage) or day 5 (blastocyst stage) of embryonic development. Luteal support consisted of a daily dose of 90 mg of progesterone in a sustained-release vaginal gel (Crinone, 90 mg; Merck Serono Limited, Switzerland) combined with oral dydrogesterone (Duphaston**, 10 mg**; Abbott B.V., The Netherlands) at a dose of 10 mg three times per day. All the medications were continued until 10–11 weeks of gestation.

### 2.4 ART outcome measures

Serum human chorionic gonadotropin (β-hCG) levels were assessed in all patients on the 14th day after embryo transfer, and biochemical pregnancy was defined as a β-hCG level >5 IU/L. Clinical pregnancy was confirmed if a gestational sac was visualized via ultrasound on the 35th day after embryo transfer.

Medical and laboratory data on patients’ characteristics and embryology were collected, including patients’ age, BMI, duration and cause of subfertility, female serum AMH level, duration and total dosage of gonadotropin stimulation, semen parameters, number of embryos transferred, implantation rate, clinical pregnancy rate (CPR), miscarriage rate, and neonatal birth weight (22).

### 2.5 Statistical analysis

SPSS statistical software (version 27.0) was used for data analysis. Categorical variables were compared between groups using Pearson’s chi-square test. Two-sided t-tests were used to assess differences in continuous variables with normal distribution, whereas non-normally distributed variables were compared using the Wilcoxon rank-sum test.

A multivariable logistic regression model was adjusted for T2DM (yes or no), duration of T2DM (continuous), fasting glucose (continuous), semen volume (continuous), progressive motility (a + b) (continuous), and asthenozoospermia (yes or no) to identify and determine the factors influencing factors of clinical pregnancy. Associations were reported as adjusted odds ratios (ORs) with 95% confidence intervals (CIs). P-values <0.05 were considered statistically significant.

## 3 Results

### 3.1 Baseline characteristics

In the initial screening, 129 patients were included in the T2DM group, and 6,741 patients were included in the control group. Of those in the T2DM group, 106 couples were ultimately included, while 23 couples canceled ART treatment. In the control group, 212 infertile couples were included and were matched 1:2 with the T2DM group according to male age, male BMI, female age, female BMI, and female AMH levels.

There were no statistically significant differences between the two groups in age, BMI, years of infertility, cause of subfertility, AMH level, basal estradiol level, FSH (follicle-stimulating hormone) level, or LH (luteinizing hormone) level of the matched patients, and the differences between these two groups (P > 0.05), as shown in **Supplementary Table 1**.

### 3.2 Comparison of semen quality between the two groups

Eight semen samples obtained by testicular needle aspiration in the T2DM group were excluded due to complete retrograde ejaculation and the absence of viable sperm in the post-ejaculatory urine. There were no significant differences between the two groups in terms of age, BMI, or smoking or alcohol use (P > 0.05) (**Table 1**).

**Table 1:** Seminal characteristics in T2DM -positive men and negative controls.

	T2DM group (n=98)	Control group (n=212)	t/x^2^/Z	P value
Baseline characteristics				
Male age (years)	37.2 ± 4.9	36.7 ± 4.9	0.786	0.432^a^
Male BMI (kg/m^2^ )	26.9 ± 4.3	26.7 ± 4.2	-0.301	0.764^b^
Duration of diabetes (years)	4.4 ± 3.4	Nil	–	–
Fasting glucose (mmol/L)	8.0 ± 1.4	5.3 ± 0.2	-13.967	**0.001^b^**
Male smoking history (n, %)			0.144	0.704^c^
Yes	23/98 (23.5%)	54/212 (25.5%)		
No	75/98 (76.5%)	158/212 (74.5%)		
Male alcohol history (n, %)			1.285	0.257^c^
Yes	25/98 (25.5%)	42/212 (19.8%)		
No	73/98 (74.5%)	170/212 (80.2%)		
Semen volume (ml)	2.4 ± 2.2	2.7 ± 1.2	-3.489	**0.001^b^**
Sperm concentration (10^6^/mL)	52.8 ± 31.0	53.0 ± 27.5	-0.056	0.955^a^
Rapid progressive motility (a) (%)	12.8 ± 11.3	16.8 ± 10.8	-2.843	**0.004^b^**
Slow or sluggish progressive motility (b) (%)	10.2 ± 6.5	12.9 ± 5.4	-3.209	**0.001^b^**
Progressive motility (a+b) (%)	23.0 ± 17.2	29.7 ± 15.3	-3.043	**0.002^b^**
Asthenozoospermia (n, %)				
Yes	74/98 (75.5%)	135/212 (63.7%)	4.271	**0.039^c^**
No	24/98 (24.5%)	77/212 (36.3%)		
Normal sperm morphology (%)	7.4 ± 3.7	8.1 ± 3.6	-1.343	0.179^b^

Values are presented as the mean ± standard deviation.

P-values were obtained using a) a Two-sided t-test, b) a Wilcoxon rank sum test, or c) a Chi-squared test. t, Two-sided t-test; x^2^, Chi-squared test; Z, Wilcoxon rank sum test.

T2DM, type 2 diabetes mellitus; BMI, body mass index.

Bold values means they are statistically significant.

Semen volume (2.4 ± 2.2 vs. 2.7 ± 1.2, P< 0.01) and the percentage of progressive sperm motility (23.0 ± 17.2 vs. 29.7 ± 15.3, P<0.01) were significantly lower in the T2DM group than in the control group. The proportion of asthenozoospermia was higher in the T2DM group than in the control group (P < 0.05). Differences in sperm concentration and the percentage of normal morphology were not statistically significant (P > 0.05).

### 3.3 Comparison of ovulation stimulation process and ART outcomes in IVF cycles between the two groups

There were 42 IVF cycles in the T2DM group and 105 IVF cycles in the control group. Gn dosage, duration of Gn stimulation, estradiol level, progesterone level, LH level, and endometrial thickness on the day of hCG injection did not differ significantly between the two groups (P > 0.05) (**Table 2**).

**Table 2:** Ovarian stimulation variables and IVF results of T2DM-positive and T2DM-negative groups.

	T2DM group (n=42)	Control group (n=105)	t/x^2^/Z	P value
Baseline characteristics				
Male age (years)	38.2 ± 4.4	37.7 ± 4.4	0.627	0.532^a^
Male BMI (kg/m^2^ )	28.2 ± 4.5	27.3 ± 4.4	1.072	0.285^a^
Female age (years)	35.5 ± 3.9	35.2 ± 3.4	0.146	0.707^a^
Female BMI (kg/m^2^ )	23.1 ± 2.9	23.5 ± 2.7	-0.719	0.473^a^
Duration of subfertility (years)	4.6 ± 3.7	5.5 ± 3.6	-1.868	0.062^b^
Cause of subfertility (n, %)				
Male factor	2/42 (4.8%)	7/105 (6.7%)	0.189	0.663^c^
Tuboperitoneal factor	17/42 (40.5%)	34/105 (32.4%)	0.868	0.352^c^
Anovulation	3/42 (7.1%)	15/105 (14.3%)	1.424	0.233^c^
Ovarian reserve dysfunction	8/42 (19.0%)	14/105 (13.3%)	0.770	0.380^c^
Endometriosis	2/42 (4.8%)	9/105 (8.6%)	0.629	0.428^c^
Unexplained	10/42 (23.8%)	26/105 (24.8%)	0.015	0.903^c^
Total dose of gonadotropin (IU)	2443.4 ± 963.3	2445.1 ± 237.4	-0.268	0.789^b^
Duration of gonadotropin stimulation (days)	10.0 ± 1.8	10.1 ± 1.9	-0.722	0.470^b^
Serum E_2_ level on the day of hCG injection (pg/ml)	2561.4 ± 1924.1	2880.8 ± 1863.2	-1.209	0.227^b^
Serum P level on the day of hCG injection (ng/ml)	0.8 ± 0.3	0.8 ± 0.4	-1.412	0.158^b^
Serum LH level on the day of hCG injection (mIU/ml)	2.3 ± 2.8	2.1 ± 1.8	-0.135	0.893^b^
Endometrial thickness on the day of hCG injection (mm)	9.1 ± 2.1	9.7 ± 2.6	-1.559	0.119^b^
2PN fertilization rate (%)	217/323 (67.2%)	557/853 (65.3%)	0.370	0.543^c^
High-grade embryo rate (%)	104/205 (50.7%)	243/504 (48.2%)	0.370	0.543^c^
Number of embryos transferred (n)	1.7 ± 0.4	1.8 ± 0.3	-1.055	0.291^b^
Implantation rate (%)	22/75 (29.3%)	58/195 (29.7%)	0.004	0.947^c^
Clinical pregnancy rate (%)	16/42 (38.1%)	48/105 (45.7%)	0.708	0.400^c^
Miscarriage rate (%)	2/16 (12.5%)	5/48 (10.4%)	0.053	0.817^c^
Baby weight (g)	2984.2 ± 523.0	3070.4 ± 509.4	-0.626	0.534^a^

Values are presented as the mean ± standard deviation.

P-values were obtained using a) a Two-sided t-test, b) a Wilcoxon rank sum test, or c) a Chi-squared test. t, Two-sided t-test; x^2^, Chi-squared test; Z, Wilcoxon rank sum test.

IVF, *in vitro* fertilization; T2DM, type 2 diabetes mellitus; BMI, body mass index; E_2_, estradiol; P, progesterone; LH, luteinizing hormone; hCG, human chorionic gonadotropin; 2PN, two-pronuclear.

Although the clinical pregnancy rate (45.7% vs. 38.1%, P>0.05) in IVF-ET cycles of the control group was higher (45.7% vs. 38.1%), the difference was not statistically significant (P > 0.05). There were also no significant differences in the 2PN fertilization rate, high-grade embryo rate, implantation rate, miscarriage rate, or neonatal birth weight (P > 0.05).

After adjusting for potential confounders—including T2DM, duration of T2DM, fasting glucose, semen volume, progressive motility (a + b), and asthenozoospermia—in the multivariable logistic regression model, no independent risk factors for clinical pregnancy were identified (P > 0.05) (**Table 3**).

**Table 3:** Logistic regression analysis of clinical pregnancies in the IVF cohort.

	Regression coefficient (β)	Standard error (SE)	P value	OR	95% CI
Variables					
T2DM	0.086	0.808	0.916	1.089	0.223-5.312
Duration of T2DM	-0.218	0.129	0.091	0.804	0.625-1.035
Fasting glucose	0.243	0.217	0.263	1.275	0.833-1.952
Semen volume	0.117	0.139	0.399	1.124	0.856-1.476
Progressive motility (a+b)	0.012	0.024	0.586	1.013	0.966-1.063
Asthenozoospermia	-0.482	0.515	0.349	0.617	0.225-1.694

IVF, *in vitro* fertilization; T2DM, type 2 diabetes mellitus; OR, odds ratio; CI, Confidence Interval.

### 3.4 Comparison of ovulation stimulation process and ART outcomes in ICSI cycles between the two groups

The T2DM group included 64 ICSI cycles, and the control group included 107 ICSI cycles. The 2PN fertilization rate, high-grade embryo rate, and implantation rate in the ICSI-ET cycles of the T2DM group were slightly lower in the T2DM group than those in the control group (76.7% vs. 78.2%; 52.7% vs. 57.9%; and 28.2% vs. 31.8%, respectively; P > 0.05), but none of these differences were statistically significant. The clinical pregnancy rate, miscarriage rate, and neonatal birth weight did not differ significantly either (P>0.05) (**Table 4**).

**Table 4:** Ovarian stimulation variables and ICSI results in the T2DM-positive and T2DM-negative groups.

	T2DM group (n=64)	Control group (n=107)	t/x^2^/Z	P value
Baseline characteristics				
Male age (years)	36.0 ± 5.2	35.7 ± 5.2	-0.226	0.822^a^
Male BMI (kg/m^2^ )	25.8 ± 3.8	26.2 ± 3.9	-0.573	0.567^a^
Female age (years)	33.5 ± 3.8	33.1 ± 3.6	-0.779	0.436^a^
Female BMI (kg/m^2^ )	22.7 ± 3.2	22.4 ± 2.5	-0.297	0.767^a^
Duration of subfertility (years)	4.8 ± 3.2	5.1 ± 3.3	-0.822	0.411^a^
Cause of subfertility (n, %)				
Male factor	29/64 (45.3%)	42/107 (39.3%)	0.606	0.436^b^
Tuboperitoneal factor	12/64 (18.8%)	28/107 (26.2%)	1.230	0.267^b^
Anovulation	10/64 (15.6%)	8/107 (7.5%)	2.823	0.093^b^
Ovarian reserve dysfunction	6/64 (9.4%)	11/107 (10.3%)	0.037	0.848^b^
Endometriosis	3/64 (4.7%)	6/107 (5.6%)	0.068	0.794^b^
Unexplained	4/64 (6.3%)	12/107 (11.2%)	1.164	0.281^b^
Total dose of gonadotropin (IU)	2373.2 ± 943.9	2406.4 ± 915.4	-0.291	0.771^a^
Duration of gonadotropin stimulation (days)	10.3 ± 1.8	10.7 ± 1.8	-1.168	0.243^a^
Serum E_2_ level on the day of hCG injection (pg/ml)	3080.5 ± 1719.1	3332.1 ± 1668.9	-0.943	0.347^c^
Serum P level on the day of hCG injection (ng/ml)	0.9 ± 0.4	0.8 ± 0.3	-0.301	0.763^a^
Serum LH level on the day of hCG injection (mIU/ml)	1.9 ± 2.9	1.5 ± 1.2	-0.868	0.385^a^
Endometrial thickness on the day of hCG injection (mm)	10.4 ± 2.7	10.2 ± 2.2	-0.291	0.771^a^
2PN fertilization rate (%)	386/503 (76.7%)	727/930 (78.2%)	0.386	0.534^b^
High-grade embryo rate (%)	157/298 (52.7%)	355/613 (57.9%)	2.226	0.136^b^
Number of embryos transferred (n)	1.9 ± 0.2	1.8 ± 0.3	-1.244	0.213^a^
Implantation rate (%)	35/124 (28.2%)	64/201 (31.8%)	0.473	0.492^b^
Clinical pregnancy rate (%)	30/64 (46.9%)	50/107 (46.7%)	0.000	0.985^b^
Miscarriage rate (%)	3/30 (10.0%)	6/50 (12.0%)	0.075	0.784^b^
Baby weight (g)	2990.0 ± 640.0	3095.5 ± 538.6	-0.815	0.418^c^

Values are presented as the mean ± standard deviation.

P-values were obtained using a) a Two-sided t-test, b) a Wilcoxon rank sum test, or c) a Chi-squared test. t, Two-sided t-test; x^2^, Chi-squared test; Z, Wilcoxon rank sum test.

ICSI, intracytoplasmic sperm injection; T2DM, type 2 diabetes mellitus; BMI, body mass index; E_2_, estradiol; P, progesterone; LH, luteinizing hormone; hCG, human chorionic gonadotropin; 2PN, two-pronuclear.

After adjusting for potential confounders—including T2DM, duration of T2DM, fasting glucose, semen volume, progressive motility (a+b), and asthenozoospermia—no independent risk factors for clinical pregnancy were identified (P>0.05) (**Table 5**).

**Table 5:** Logistic regression analysis of clinical pregnancies in the ICSI cohort.

	Regression coefficient (β)	Standard error (SE)	P value	OR	95% CI
Variables					
T2DM	-0.392	0.846	0.644	0.676	0.129-3.549
Duration of T2DM	0.164	0.090	0.070	1.178	0.987-1.407
Fasting glucose	-0.461	0.241	0.056	0.631	0.393-1.011
Semen volume	0.150	0.125	0.231	1.162	0.909-1.485
Progressive motility (a+b)	0.010	0.140	0.479	1.010	0.982-1.039
Asthenozoospermia	-0.119	0.609	0.845	0.888	0.269-2.962

ICSI, intracytoplasmic sperm injection; T2DM, type 2 diabetes mellitus; OR, odds ratio; CI, Confidence Interval.

## 4 Discussion

The proportion of male factors contributing to infertility has been gradually increasing, potentially due to environmental factors, work stress, and modern lifestyles, including the widespread consumption of high-fat and high-sugar foods. DM is one of the chronic diseases that seriously jeopardizes public health. More than 500 million people worldwide are affected, the vast majority of whom have T2DM. Metabolic syndrome (MetS) represents a cluster of conditions that include impaired glucose metabolism, hypertriglyceridemia, hypertension, low HDL cholesterol, and abdominal obesity; these identify subjects at high risk of developing T2DM and cardiovascular diseases. Emerging evidence suggests that MetS may negatively impact male reproductive potential, implying a potential association between T2DM and male fertility (23). However, the impact of T2DM on male reproductive function has only recently come into focus. Animal studies have shown that diabetes reduces the fertility of male animals, while clinical studies have demonstrated that diabetes leads to decreased sperm quality, male erectile dysfunction, ejaculatory dysfunction, hypogonadotropic hypogonadism, and delayed puberty (24, 25). For instance, men with DM and erectile dysfunction exhibit a worse metabolic profile that is associated with poor semen quality, compared to those without erectile dysfunction (26). Some studies have shown that semen volume and the percentage of forward-moving spermatozoa (including rapid progressive motility and slow or sluggish progressive motility) are significantly lower in patients with T2DM compared to controls (P < 0.05). Although sperm concentration and the percentage of normal morphology spermatozoa were lower in the T2DM group, these differences were not statistically significant. These findings suggest that T2DM negatively affects semen quality and male fertility (24, 25). The effect of T2DM on sperm volume, concentration, motility, and morphology in humans remains controversial at present. Ali et al. compared 314 male patients with T2DM to 100 healthy individuals and found that the T2DM group had lower semen volume and reduced sperm viability, with no significant difference in sperm concentration or normal morphology between the two groups (27). Rama Raju et al. compared 35 male patients with T2DM to 123 healthy controls and found that sperm viability was lower in the T2DM group, with no differences in other semen parameters (18). In contrast, Inih et al. evaluated 108 male patients with T2DM and 66 controls and demonstrated lower total sperm count, viability, and normal morphology in the T2DM group (28). A meta-analysis conducted in 2016 reported that T2DM appeared to decrease seminal volume and sperm motility in men who were undergoing infertility screening; however, it did not affect total sperm count or the percentage of normal sperm morphology (29). A 2023 review similarly reported a negative effect of T2DM on semen volume, sperm motility, and normal sperm morphology, with no effect on sperm concentration or total count (30). These discrepancies may be related to the fact that diabetes affects semen parameters (consisting of spermatozoa and seminal plasma) through various pathways. In our study, the T2DM group showed lower semen volume, reduced sperm motility, and a higher incidence of asthenozoospermia compared to the control group (P < 0.05), which reflected that T2DM might impair semen quality. Spermatogenesis is a time-consuming and multifactorial physiological process involving multiple tissues and organs, and it is accomplished by the coordinated regulation of nerves, endocrine hormones, and multilayered effects. Any process of the body’s neural or endocrine systems affected by diabetes can result in changes in semen quality (11, 14, 15): 1) Hyperglycemic exposure leads to changes in the hypothalamic GnRH pulse release, affecting LH and FSH production. This, in turn, impacts the function of the seminiferous tubules and testicular cells, thereby influencing spermatogenesis. 2) Chronic inflammation associated with T2DM can affect the endocrine axis at all levels, inducing alterations in gonadal hormone production at the hypothalamic-pituitary level and triggering changes in sperm membrane and functional development at the testicular level. 3) Diabetic microvascular and neuropathic complications can cause erectile and ejaculatory dysfunction, leading to impaired semen emission and consequently affecting semen volume.

Overall, there is no doubt that diabetes affects male semen quality and, consequently, the natural fertility of men, regardless of the mechanism acting on the male reproductive system.

Relevant investigations have revealed infertility rates of 35.1% to 51% in male subjects with diabetes (16). Therefore, with the advancement of ART, an increasing number of male diabetic patients are opting to fulfill their desire for fatherhood through IVF or ICSI. However, there remain numerous uncertainties regarding the outcomes of these reproductive efforts.

Currently, few articles have been published on outcome studies of male diabetic patients undergoing ART, except for two early studies. In 2011, Mulholland et al. evaluated the potential relationship between male diabetic patients and IVF/ICSI outcomes. This research was based on 80 couples in which the male partner had diabetes, including 18 who applied for ART (5 chose IVF, 12 chose ICSI, and 1 chose both). Fertilization rates (68% for IVF and 62% for ICSI) were similar to those of non-diabetic patients (70% for IVF and 71% for ICSI), with no difference in embryo quality observed. Following the embryo transfer, one female diabetic patient exhibited a pregnancy rate of 5%, which was lower than anticipated (28.8%). Among the seven patients who underwent frozen embryo transfers, there were two successful pregnancies (28.6%), which was higher than the expected rate (21.3%). Overall, there was no difference in fertilization rates and embryo quality between couples with and without a male diabetic partner. However, couples with male diabetic partners exhibited a lower pregnancy rate (17). Raju et al. conducted a study in 2012 comparing the semen characteristics and sperm DFI of 35 male patients with T2DM treated with ART against 123 male non-diabetic subjects. They correlated these parameters with pregnancy outcomes. The study revealed that T2DM patients exhibited lower progressive (Type A) sperm motility (14.64 ± 9.60 vs. 17.99 ± 11.51, P < 0.02), and other semen parameters were similar to those of the control group (which is similar to our findings). Furthermore, the blastocyst formation rate was lower in the T2DM group undergoing IVF compared to the control group (38.13% vs. 55.46%, P < 0.001), as was the pregnancy rate (28.57% vs. 55.46%, P < 0.001). Additionally, the miscarriage rate was higher in the T2DM group (50.0% vs. 24.56%, P < 0.001). Sperm damage may act as a mediator linking T2DM to adverse ART outcomes. The above findings suggest that male diabetic patients may negatively impact ART outcomes (18). Nonetheless, our results did not reflect the findings of the two earlier studies mentioned above. This study compared 106 transfer cycles in the T2DM group with 212 transfer cycles in the healthy group. The results indicated that the differences in the 2PN rate, high-grade embryo rate, embryo implantation rate, clinical pregnancy rate, miscarriage rate, and neonatal weight were not statistically significant between the T2DM group and the control group (P > 0.05). We were able to include a larger study population and a multiple-factor analysis of male T2DM in IVF/ICSI cycles. This enabled us to investigate whether male T2DM influences the likelihood of successful pregnancy, and reassuringly, our results did not show this effect.

These findings imply that the latest techniques for the preparation and selection of human spermatozoa may enhance the success rates of fertilization capacity, embryo production, and *in vitro* survival, and pregnancy and delivery rates in patients undergoing IVF/ICSI treatment (31). The extent to which initial sperm quality affects ART success rates may be due to the bypassing of *in vivo* sperm selection by both IVF and ICSI. For instance, research has shown that the addition of creatine to the culture medium prolongs sperm capacitation, thereby achieving successful fertilization of 60% of the oocytes (32). While sperm-derived defects, such as DNA damage and epigenetic abnormalities, may not prevent fertilization, they can result in early developmental arrest, poor blastocyst formation, or increased aneuploidy. The *in vitro* culture and blastocyst morphologic score systems function as a selection process, with embryos that reach the blastocyst stage having presumably overcome or corrected these paternal defects, thereby compensating for initial sperm factors and improving IVF outcomes (33). Asthenozoospermia is a cause of infertility. In its severe form, a dramatic decrease or absence of sperm motility in the ejaculate makes it impossible for the sperm cell to autonomously reach and fertilize the oocyte. ICSI represents the best option to achieve fertilization, embryo development, and live births (34). This is reflected in our data, which show that severe asthenozoospermia cases are more commonly encountered in ICSI cycles.

Over the years, as the field of ART has continued to evolve, personalized ART treatment based on the genetic and epigenetic characteristics of patients and embryos has greatly improved the safety and outcomes of ART, supported by the increasingly widespread use of genomic technology and bioinformatics. This progress is mainly reflected in the following two aspects. On the one hand, technological advancements—such as enhanced laboratory culture technology, improvements in culture media, and refinements in fertilization techniques—have greatly improved embryo culture conditions. On the other hand, refinements in clinical treatment protocols, in which clinicians develop individualized ovulation stimulation plans and embryo transfer strategies based on patients’ medical conditions, have optimized clinical decision-making and techniques to further enhance the success rates of ART treatment (35, 36). These studies and the ongoing refinement of clinical treatment strategies are essential, as they can enhance the fertility rates of patients with various types of infertility.

This study is the first to examine the impact of male patients with T2DM on the outcome of IVF/ICSI cycles. Analysis of the data reveals that T2DM affects male semen parameters; however, the decline in sperm quality does not translate into compromised clinical ART outcomes, which contradicts the conclusions of two previous studies. Obviously, numerous other unknown mechanisms may contribute to diabetes-related reproductive dysfunction and require further exploration in future research. As a retrospective study, however, there are several limitations. The major limitations are the predetermined calculation of the sample size, incomplete medical records, and the lack of detailed information on male patient characteristics, such as HbA1c values and hormonal levels (e.g., LH, FSH, testosterone) in our dataset; these factors may also influence fertility outcomes in ways yet to be discovered. In addition, one of the vital indicators associated with diabetes and sperm DNA damage—DFI—was not included, as more than half of the data were missing. Due to the sample size and incomplete DFI data, we were challenged by insufficient statistical power. It is hoped that future studies may consider adopting a larger sample size and more detailed medical data records with follow-up in a multicenter prospective manner.

In conclusion, this study demonstrates that T2DM negatively impacts male semen quality but does not translate into compromised clinical ART outcomes, which emphasizes the importance of ART treatment in protecting fertility and equal reproductive rights for male patients with T2DM. With the advancement of ART and personalized treatment strategies, pregnancy outcomes are expected to improve, thereby enabling an increasing number of male patients with T2DM experiencing infertility to fulfill their reproductive desires through these methods.

## Data Availability

The raw data supporting the conclusions of this article will be made available by the authors, without undue reservation.
