# Supplementary material for: Effect of type 2 diabetes mellitus on sperm quality and outcomes of assisted reproductive techniques in infertile male patients
Source: Front Endocrinol (Lausanne). 2026 Jan 5;16:1692746. doi: 10.3389/fendo.2025.1692746 (PMC12812604; doi:10.3389/fendo.2025.1692746)
Supplement: Supplementary file 1 [file Table1.docx]

Supplementary Table 1. Baseline characteristics of the study population.

|  | T2DM  （n=106） | Control  （n=212） | t/x^2^/Z | P value |
| --- | --- | --- | --- | --- |
| Female age (years) | 34.3±4.0 | 34.1±3.7 | 0.239 | 0.811^a^ |
| Female BMI (kg/m^2^ ) | 22.9±3.1 | 23.0±2.7 | -0.422 | 0.659^b^ |
| Duration of subfertility (years) | 4.7±3.4 | 5.3±3.4 | -1.925 | 0.054^b^ |
| Cause of subfertility (n, %) |  |  |  |  |
| Male factor | 31/106 (29.2%) | 49/212 (23.1%) | 1.411 | 0.235^c^ |
| Tuboperitoneal factor | 29/106 (27.4%) | 62/212 (29.2%) | 0.123 | 0.726^c^ |
| Anovulation | 13/106 (12.3%) | 23/212 (10.8%) | 0.141 | 0.707^c^ |
| Ovarian reserve dysfunction | 14/106 (13.2%) | 25/212 (11.8%) | 0.132 | 0.717^c^ |
| Endometriosis | 5/106 (4.7%) | 15/212 (7.1%) | 0.667 | 0.414^c^ |
| Unexplained | 14/106 (13.2%) | 38/212 (17.9%) | 1.150 | 0.284^c^ |
| Basal AMH (ng/ml) | 3.8±3.3 | 3.7±3.2 | -0.255 | 0.799^b^ |
| Basal E_2_ (pg/ml) | 35.0±16.4 | 34.5±17.7 | -0.425 | 0.671^b^ |
| Basal FSH (mIU/ml) | 6.0±1.9 | 6.1±1.8 | -0.328 | 0.743^b^ |
| Basal LH (mIU/ml) | 3.4±1.7 | 3.5±1.6 | -0.421 | 0.674^b^ |

Values were presented as mean ± standard deviation

P-values were obtained using a Two-sided t-test, b Wilcoxon rank sum test, or c Chi-squared test.

T2DM, type 2 diabetes mellitus; BMI, body mass index; AMH, anti-Müllerian hormone; E_2_, estradiol; FSH, follicle stimulating hormone; LH, luteinizing hormone.
